# Advances of surgical robotics: image-guided classification and application

**DOI:** 10.1093/nsr/nwae186

**Published:** 2024-06-06

**Authors:** Changsheng Li, Gongzi Zhang, Baoliang Zhao, Dongsheng Xie, Hailong Du, Xingguang Duan, Ying Hu, Lihai Zhang

**Affiliations:** School of Mechatronical Engineering, Beijing Institute of Technology, Beijing 100081, China; Department of Orthopedics, Chinese PLA General Hospital, Beijing 100141, China; Shenzhen Institute of Advanced Technology, Chinese Academy of Sciences, Shenzhen 518055, China; School of Mechatronical Engineering, Beijing Institute of Technology, Beijing 100081, China; School of Medical Technology, Beijing Institute of Technology, Beijing 100081, China; Department of Orthopedics, Chinese PLA General Hospital, Beijing 100141, China; School of Mechatronical Engineering, Beijing Institute of Technology, Beijing 100081, China; School of Medical Technology, Beijing Institute of Technology, Beijing 100081, China; Shenzhen Institute of Advanced Technology, Chinese Academy of Sciences, Shenzhen 518055, China; Department of Orthopedics, Chinese PLA General Hospital, Beijing 100141, China; Shenzhen Institute of Advanced Technology, Chinese Academy of Sciences, Shenzhen 518055, China

**Keywords:** medical robotics, surgical robotics, medical image processing, robot-assisted surgery

## Abstract

Surgical robotics application in the field of minimally invasive surgery has developed rapidly and has been attracting increasingly more research attention in recent years. A common consensus has been reached that surgical procedures are to become less traumatic and with the implementation of more intelligence and higher autonomy, which is a serious challenge faced by the environmental sensing capabilities of robotic systems. One of the main sources of environmental information for robots are images, which are the basis of robot vision. In this review article, we divide clinical image into direct and indirect based on the object of information acquisition, and into continuous, intermittent continuous, and discontinuous according to the target-tracking frequency. The characteristics and applications of the existing surgical robots in each category are introduced based on these two dimensions. Our purpose in conducting this review was to analyze, summarize, and discuss the current evidence on the general rules on the application of image technologies for medical purposes. Our analysis gives insight and provides guidance conducive to the development of more advanced surgical robotics systems in the future.

## Teaser

Novel classification of the guidance images applied for surgical robotics is proposed, and the current surgical robotics in different application scenarios are analyzed, directing researches to fill the sensing gaps.

## INTRODUCTION

In the past decades, the field of surgical robotics has gained momentum, and thousands of robotic surgical systems have now been installed in clinics around the world, which have performed millions of procedures [1]. As advanced medical equipment, surgical robots can assist surgeons to complete minimally invasive, accurate, and efficient operation, which can bring only a small amount of trauma, alleviate patients’ pain, shorten postoperative recovery time, and augment surgical ability [2–4]. These advantages have been attracting increasingly more global research attention, especially in the field of minimally invasive surgery, with huge market scale and broad application prospects [5–7].

Surgical robotics encompasses perception, control, and actuation units. The fundamental execution logic of the operating system is to establish the correlation and mapping between different spaces, including image data space, patient physical space, robot motion space, etc., regardless of whether it is a positioning robot with an autonomous planning module, an operating robot using the master-slave control method, or even an operating robot that can perform autonomous operations in the future [8–11].

Compared to other perceptive modes, human environmental understanding and memory primarily rely on vision [12,13]; image-guided robot vision plays a similar role [14]. Images serve as a primary source of robot environment perception, which provides intuitive guidance for surgical procedures and improve the efficiency and accuracy of surgery [15–18]. Through image processing algorithms, such as data augmentation, object segmentation, and instrument tracking, robots can understand the environment and respond quickly and accurately [19–24]. Combined with the inherent advantages of robots, including dexterous and fine operation, surgical robotic systems can provide more possibilities for the development and breakthroughs in the operating room, promoting the development and advancement of minimally invasive, intelligent, and automatic surgery [25–28].

Different types of surgical robots employ distinct methods for image acquisition, spatial system establishment methods, and application modes, even within the same category. Traditional analysis and application of image information is often based on the image source, which lacks systematic classification and sorting. Consequently, research often emphasizes image sharpness and analytic power such as image super-dimension and enhancement, without offering a comprehensive understanding of the image application from the macroscopic perspective of surgical robotics. This paper aims to summarize spatial localization and target-tracking methods based on images, to determine the conventional practice of the application of surgical robotics, and propose an image-guided dual-cross classification standard that can provide guidance for future advancement in this field.

The searches were developed and conducted by a trained researcher, BLZ, in evidence synthesis searching. The researcher developed a search strategy using subject headings and keywords for the following databases: PubMed, SpringerLink, Elsevier, IEEE Xplore and Scopus. The strategies were peer-reviewed by another researcher, LHZ, trained in evidence synthesis searching. All database results were exported to EndNote, duplicates were removed using a multi-step process. Keywords were determined according to different content such as specified names of medical imaging technologies, names of medical image processing technologies and names of robotic systems. The core screening criterion was related to image-guided robot-assisted surgery. Only English-language papers were considered for inclusion; case reports, conference abstracts, letters to the editor, editorials, and reviews were excluded. Two independent authors (BLZ and ZGZ) conducted a thorough screening of all retrieved records based on the titles and abstracts, and then full texts were retrieved and reviewed for eligibility. In cases of discrepancies, a discussion was held to reach a resolution. The full texts of the screened papers were further assessed for eligibility, and those remaining were included in the present review. The three processes of identification, screening, and selection of scientific articles to be included in our review were supervised by an experienced author (LHZ). A total of 235 studies were chosen after two stages of screening. Most of the selected studies were in the last two decades (217 studies in 2004–2023), while some technological definitions could be traced back to 1982.

The main contributions of this paper are listed as follows:

1. A novel classification of the guidance images applied for surgical robotics is proposed, which categorizes imaging technologies into direct and indirect based on the acquisition object, and into continuous, intermittent continuous, and discontinuous based on the target-tracking frequency.
2. From the two guidance image classification dimensions, the current surgical robotics in different application scenarios are analysed, which helps to simplify the robot selection and direct the research to fill the sensing gaps.
3. A novel categorization of the existing surgical robotics operation into ‘human logic’ and ‘robot logic’ is proposed, and it is suggested to combine them with intelligence and automation improvement.

## IMAGING TECHNOLOGIES RELATED TO SURGICAL ROBOTICS

Images in the field of surgical robotics can be acquired through non-invasive medical imaging techniques: computed tomography (CT), magnetic resonance imaging (MRI), radiography, ultrasonography, etc. In addition, the preoperative 3D model and navigation images can be obtained through sensors, such as visual, optical, and electromagnetic sensors [29–32]. This section details the concepts, advantages, risks, and applications of these technologies.

### Image-guided dual-cross classification

**Figure 1.**
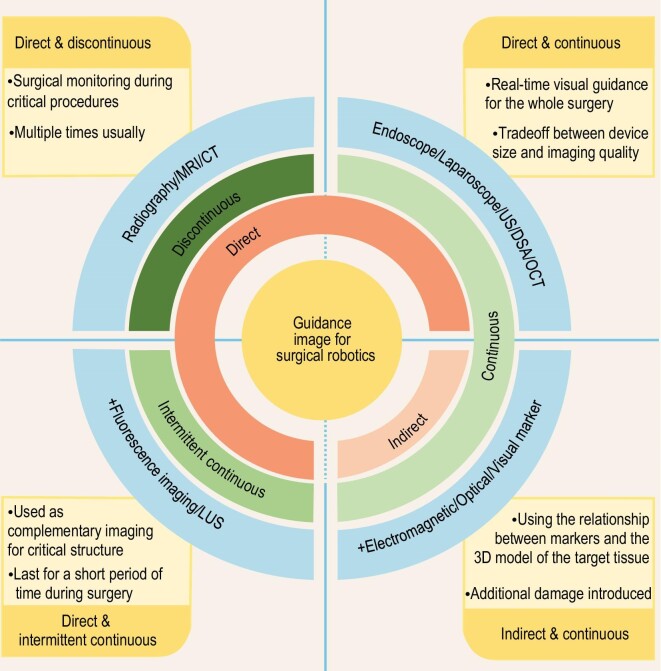
Classification of the guidance image applied for surgical robotics.

As illustrated in Fig. 1, we categorized imaging technologies into direct and indirect based on the acquisition object, and into continuous, intermittent continuous, and discontinuous based on the target-tracking frequency. The direct/indirect classification refers to the image's capacity to display the morphological and anatomical characteristics of the target tissue, directly correlating to the accuracy and reliability of the information obtained. On the other hand, the continuous/intermittent continuous/discontinuous classification is based on the utilization frequency of the image, typically determined by the human eye's perception of the image which is usually accepted as a dividing line. This category is primarily influenced by the image acquisition and processing rates. Meanwhile, the frequency of the use of imaging technologies that cause damage to living tissues should be minimized as much as possible. We established the proposed classification system based on the aforementioned two dimensions, which can summarize the applications of various imaging techniques accurately and comprehensively. Subsequently, we constructed a graph of imaging applications for surgical robotics.

#### Direct image

A direct image refers to an image that can reflect the morphology and characteristics of the target tissue directly and can be directly applied in robots. For example, real-time images of the target area can be obtained through a laparoscopic camera in the slave end and displayed on a screen to guide surgical operations using master-slave robotic systems [31]. This method offers real-time and intuitive information but is limited to acquiring surface features without penetrating the tissue's interior, resulting in a narrow field of vision. Intraoperative ultrasound can obtain images of the inside of the tissue and can thus explore a wider range of tissues than laparoscopic cameras [33,34]. However, it is characterized by poor image quality, especially in the case of soft tissue contrast. In addition, extrusion deformation in the soft tissue is caused by the squeeze of ultrasound equipment, affecting the accuracy. Radiography can illustrate the position of the surgical tool and the target area during the operation, which is often used for intraoperative navigation and positioning. However, this method emits radiation to both patients and surgeons and should be used sparingly [35].

#### Indirect image

Indirect image refers to an image that cannot directly display the target tissue, but requires preprocessing to enhance the quality for further analysis. For example, markers can be inserted and attached to patients that can be recognized by X-ray, infrared, visible light, and other imaging methods. Subsequently, a transformation matrix is established through registration, enabling surgical robots to indirectly obtain the position of the target tissue [20,29,36]. This approach is suitable for situations where obtaining a direct image is unfeasible, as is the case with human bones, enveloped in muscle, ligament, and skin tissues, which makes their morphology challenging to discern under minimally invasive conditions. However, errors in the marker calibration and registration algorithm can arise due to the additional processing required, resulting in lower accuracy compared to direct imaging. Moreover, attaching markers increases the complexity of the process and poses additional risks of infection and discomfort for patients [37].

#### Continuous image

Continuous image refers to continuous use during an operation, such as the image provided by an endoscopic camera, laparoscopic camera, ultrasound and DSA unit [38,39]. The continuity is defined by the frequency of acquisition, usually by the continuity of the human eye's perception. The high image frequency acquisition can ensure timeliness of information.

#### Intermittent continuous image

Intermittent continuous image refers to a continuous image but only lasts for a short period of time during surgery, such as laparoscopic ultrasound and fluorescence images. These are typically used as complementary imaging for critical structures, providing additional information for improved surgical outcomes. For example, in minimally invasive abdominal surgery, the laparoscope can only provide surface information of tissue, while the laparoscopic ultrasound can provide the anatomic structure of the target tissue in depth and help to locate blood vessels. Fluorescence imaging, on the other hand, delineates tumor boundaries and different functional tissue areas, leveraging differences in vascular perfusion of contrast medium. These images are continuous but utilized only when necessary, providing complementary information to surgeons regarding the target tissue.

#### Discontinuous image

Discontinuous image indicates the presence of a certain time interval around each image acquisition. For example, a CT image used in surgical monitoring cannot be continuously acquired due to the scanning time [40]. Additionally, imaging technologies causing damage to human tissues such as radiation should be used as infrequently as possible, and thus they also belong to this category. Discontinuous imaging typically exhibits poor real-time performance, and the time gap between image acquisition and the actual surgical procedure leads to a deviation in surgical accuracy, such as displacement caused by respiratory movement and heart beating of a patient. Intraoperative compensation methods, such as taking radiography images for critical procedures, improving timeliness through 2D-3D registration, are commonly employed to improve imaging accuracy [41,42], and correcting the position of the surgical robot through optical and electromagnetic devices.

### Imaging technologies related to surgical robotics

Table 1 outlines the prevalent types and characteristics of imaging technologies in both surgical robotics and clinical practice. CT has the advantages of fast scanning speed and high resolution, but it requires ∼3–5 minutes for scanning, exhibits poor real-time performances, and delivers a high radiation dose, which can be harmful to tissues [40,43]. Conversely, MRI has the advantage of no radiation, but its spatial resolution is inferior to that of CT, and it necessitates a longer scanning time with a higher prevalence of artifacts than CT scans [44–47]. Patients with cardiac pacemakers or body parts with metallic foreign bodies cannot undergo MRI. Radiography is commonly used to monitor the progress of orthopedic surgery [48–50]. Compared with CT, it has a lower radiation dose, but the real spatial position of the target is difficult to establish, which is not intuitive for surgeons [35,51]. Ultrasonography is advantageous due to its real-time and radiation-free application, providing detailed images of internal organs, lesions, and glandular tissue anatomy. It is commonly used to display the location, size, and shape of organs, the range and physical properties of lesions, and anatomical maps of glandular tissue [33,34,52]. However, the accuracy of the images collected by ultrasound equipment depends on the propagation of ultrasound in human tissues, which causes poor contrast and repeatability, and is closely related to the operator's experience.

**Table 1.** Common guidance images and their characteristics in clinical practice.

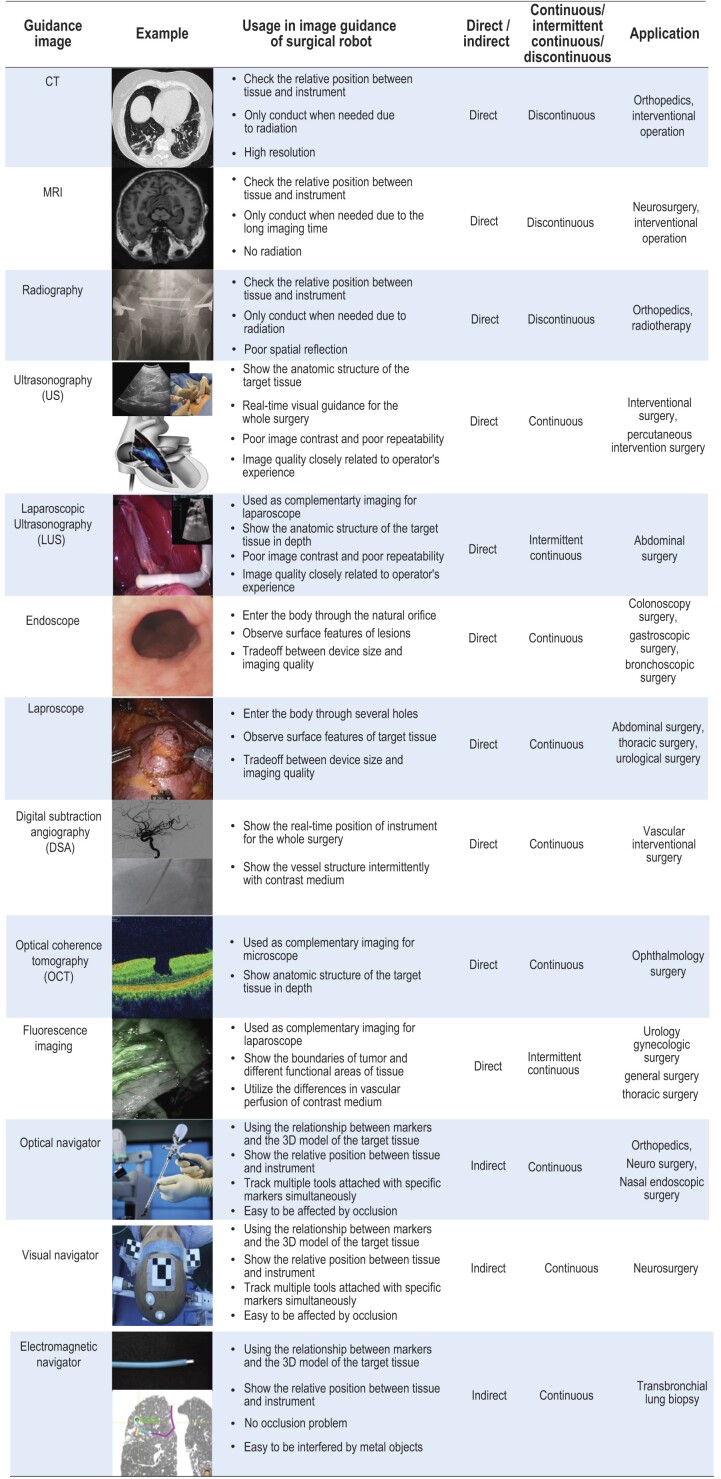

Laparoscopes and endoscopes serve as visual sensors that can enter the body to observe surface features of the target tissue [53–55]. They can be categorized into rigid lens and flexible lens depending on the lens body flexibility which can change the direction of its movement. Optical and visual navigators adopt natural light or near-infrared-ray (NIR) to track passive/active wireless markers, which can simultaneously track multiple tools attached with specific markers in a large measurement volume. While NIR navigation achieves sub-millimeter accuracy, its widespread adoption is impeded by high cost [29,56,57]. Natural light navigation has a cost advantage, but its accuracy is lower than that of NIR [58,59]. Optical navigation is an indirect tracking method, whose performance is easily affected by occlusion, and the implantation of markers causes additional damage to patients. Electromagnetic navigators generate magnetic fields in the surgical area through field generators and measure the position and pose of the induction coil in the magnetic field to realize real-time tracking. It can avoid the problem of occlusion, but it is easy to be interfered with by metal objects used during the operation, which affects the measurement accuracy [60–63].

In addition, specific imaging modalities serve distinct purposes. For instance, optical coherence tomography (OCT) is a non-contact, high-resolution tomography and biomicroscopic imaging method [64,65], which is mainly used in the field of ophthalmology [66]. It is used as complementary imaging for the microscope and has advantages such as the absence of contact, tissue destruction, and radiation; however, it requires a sufficient penetration depth for achieving high-quality imaging [67–69]. Digital subtraction angiography (DSA) has the characteristics of high contrast and resolution, which can be applied to observe vascular lesions and digitally measure vascular stenosis [70–72]. It is utilized mainly for the treatment of coronary heart disease, arrhythmia, valvular disease, and congenital heart disease. Fluorescence imaging can clearly show the boundaries of tumors and different functional areas of tissue, and can help increase the accuracy of tumor resection and decrease the blood loss for partial nephrectomy and liver resection [73,74].

### Related algorithm for image processing

**Figure 2.**
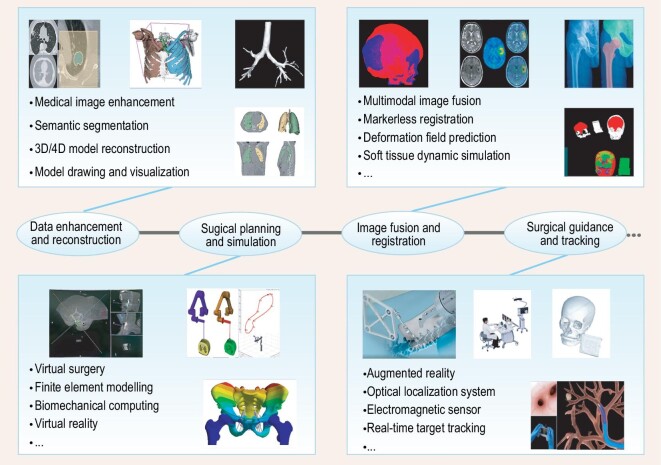
Image processing technologies in surgical robotics.

As depicted in Fig. 2, we summarized the current applications of imaging in the field of surgical robotics based on literature review, including data enhancement and reconstruction, surgical planning and simulation, image fusion and registration, and surgical guidance and tracking.

#### Data enhancement and reconstruction

Data enhancement and reconstruction includes mainly noise reduction, smoothing, resolution enhancement, and reconstruction algorithms based on the images to present a visualization result of a virtual model [19], such as radiography film splicing, three-dimensional (3D) reconstruction, and dynamic reconstruction of organs [79–83]. In clinical settings, these methods can assist surgeons in tissue visualization and surgical planning, but the reconstruction and accuracy of tiny tissues still needs to be improved [84,85]. In the field of natural orifice transluminal surgery, some researchers have adopted simultaneous localization and mapping algorithm (SLAM) in the field of computer vision (CV), which can perform 3D reconstruction of natural orifices through images collected by endoscopic cameras [86,87], and artificial intelligence makes it feasible for 3D reconstruction from monocular cameras, which reduces the cost [55,88].

#### Surgical planning and simulation

The planning of surgical procedures (such as a needle entry path and osteotomy area) using a 3D virtual model is referred to as surgical planning and simulation [89,90]. With the rapid advancements in computer graphic (CG) and VR technologies, surgeons can perform graphic calculations and surgical simulations on virtual targets [91–94], and surgeons can conduct an interactive operation on a virtual model and obtain real-time visual and haptic feedback, which can effectively improve the efficiency of surgical plan formulation and the success rate of surgery [95,96].

#### Image fusion and registration

Image fusion and registration includes the fusion of images acquired by different imaging methods [74,97–100] and calibrating among image data space, patient physical space, and robot motion space, etc. Based on data dimensions, it can be divided into 2D-2D, 2D-3D, and 3D-3D registration [101–104]. Dynamic, elastic, and markerless registrations of deformable tissues are hot topics in this field, and state-of-the-art deep learning methods can significantly improve the speed and accuracy of registration, providing technical support for intraoperative multimodal image fusion navigation [105–110,239].

#### Surgical guidance and tracking

Surgical guidance and tracking usually refers to lesion detection and segmentation, and real-time tracking of surgical targets and instruments, thus providing surgeons with guidance during surgery [9,75–78,111]. Advanced learning-based algorithms can even accomplish surgical environment understanding with surgical scene segmentation and workflow recognition [234–236], promoting autonomous robotic surgery [237,238]. For tissue deformation, some researchers have employed measurements using redundant markers/trackers to correct spatial relationships during operation [112–115], but achieving appropriate accuracy is still a big challenge. Furthermore, AR technology provides a new solution for intraoperative visual guidance, which has been applied in orthopedics, abdominal surgery, thoracic surgery, etc. [116–120].

### Imaging technologies in dual-cross classification

As shown in Fig. 1, according to the dual-dimension division of the image acquisition and target-tracking methods, the application of images can be divided into four categories: direct acquisition and discontinuous use, direct acquisition and continuous use, direct acquisition and intermittent continuous use, and indirect acquisition and continuous use. Furthermore, some surgical robotic systems utilize a combination of multiple image types.

#### Direct and discontinuous

Direct acquisition and discontinuous use of images usually adopts radiography, CT, and MRI, and is commonly implemented to intraoperatively monitor critical surgical procedures because of the time consumed and the ionizing radiation emitted. This category is particularly suitable for orthopedic surgery and puncture surgery, such as using CT and MRI images to monitor the process of puncture needle insertion and using radiography to monitor the progress of fracture reduction [49,121].

#### Direct and continuous

Direct acquisition and continuous use of images are usually achieved through laparoscopic/endoscopic or ultrasound images for minimally invasive surgery in the thoracic and abdominal cavity and natural orifice surgery [122–124]. Vascular interventional surgery guided by DSA and ophthalmology guided by microscope and OCT also belong to this category. Real-time acquisition of the pose and position of the target tissue and instruments through images facilitates accurate operations.

#### Direct and intermittent continuous

Direct acquisition and intermittent continuous use of images usually refers to laparoscopic ultrasound and fluorescence imaging. These are usually used in minimally invasive abdominal surgery as complementary imaging for laparoscopes. Since the laparoscope can only observe surface information of the target tissue, when surgeons conduct resection operations, they need laparoscopic ultrasound to show the tissue information in depth and locate blood vessels, and fluorescence imaging to show the boundaries of tumor and different functional areas of tissue, thus improving the accuracy and safety of surgery.

#### Indirect and continuous

Indirect acquisition and continuous use of images include registration and tracking two stages. Radiography, CT, and other imaging technologies are used for the registration of the image and physical spaces, while optical, electromagnetic, and other real-time navigation devices are utilized to track the position and pose of markers [114,117], indirectly enabling real-time tracking of the surgical area or target tissue. This approach enhances tracking timeliness while ensuring accuracy, although the placement of markers introduces additional damage. It is commonly used for guidance procedures in orthopedic surgery such as pedicle screw and positioning needle implantation.

In addition to the above combination methods, some complex methods have also been implemented. For example, registration through discontinuous images and tracking through continuous images has both real-time performance and high precision [36]. Multi-mode information fusion is used to avoid the deviation problems caused by the discontinuity of the single type of data [125]. The finite element method is used for biomechanical calculation to convert a static image into a dynamic image [126,127]. The environmental perception ability of surgical robots is improved by combining other sensory information [128,129].

The application of images is closely related to the target tissue and the operation method. The surgical process in environments with transparent media cavities, such as the abdominal cavity, digestive tract, and the space between joints, can be observed with high frequency and high quality by direct acquisition and intermittent continuous/continuous use [54,130,131]. For surgical procedures demanding high accuracy, direct acquisition and discontinuous use can reduce radiation doses for surgeons and patients while ensuring the accuracy of surgery [132,133]. For surgical procedures with high demands for accuracy and timeliness, the choice of indirect acquisition and continuous use can achieve a satisfactory balance between them [134–136].

## EXISTING SURGICAL ROBOTS IN IMAGE-GUIDED DUAL-CROSS CLASSIFICATION

Several classification criteria of surgical robotics have been suggested in earlier reports. For example, based on the hospital department, it can be categorized into orthopedic [137–140], neurosurgical [141–143], thoracic [122,123,144,145], or digestive surgical robotics [131,146]. Based on the target surgical operation, it can be classified into fracture reduction [147–149], arthroplasty [150,151], puncture [152–154], osteotomy [155–157], and vascular intervention surgical robotics [158–161]. Additionally, according to the application environment, it can be categorized into laparoscopic robotics [130,162,163], natural orifice transluminal robotics [164–166], etc. These classification criteria can intuitively describe a certain aspect of robots, but they cannot reveal the fundamental attributes of the classified categories, as well as the correlations and differences between different categories, making them non-universal and non-comprehensive [167]. Surgical robots vary in design, functionality, environment, and target tissue, consequently influencing the approach to imaging application.

**Figure 3.**
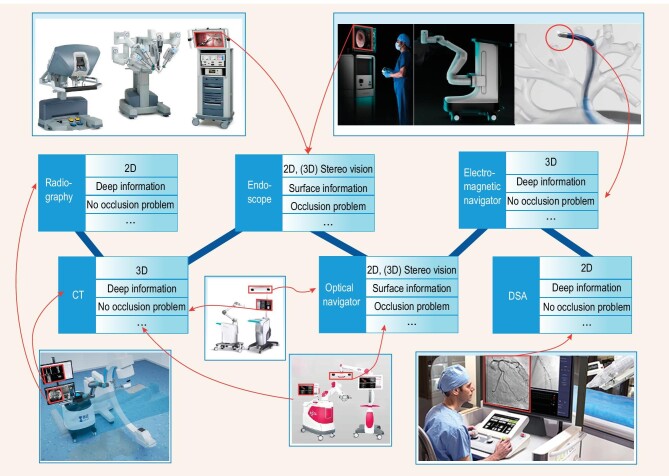
Application of imaging technologies in surgical robotics.

The image itself possesses various inherent properties, including 2D/3D, shallow/deep information, with/without an occlusion problem, as is illustrated in Fig. 3. We performed an interdisciplinary survey of the existing literature to review the current status of surgical robotics’ clinical research and application, with a focus on the robotics systems that have already been applied in clinical settings. First of all, surgical robotics applications are classified and introduced according to common surgical classification methods, such as laparoscopic surgical robotics, orthopedic surgical robotics, vascular interventional surgical robotics, puncture surgical robotics, natural orifice transluminal surgical robotics, and other surgical robotics. The application mode and characteristics of images in each category is incorporated into the classification system proposed in this article.

### Laparoscopic surgical robotics

Laparoscopic surgical robotics is currently a key focus in the field of surgical robotics, typically referring to the multi-port and single-port surgical robotics used for thoracic and abdominal surgery. This type of surgical robotics is widely used in general surgery, gynecology, urology, cardiac surgery, etc. Inflation is a common way to reduce the surgical wound and increase the movable space of the robot in abdominal surgery, while the collapse of the lung caused by the change in air pressure is employed as the surgical space in thoracic surgery. The application environment is a gas chamber, and a visual sensor can be integrated in the robotic system for real-time image acquisition, and thus direct acquisition and continuous/intermittent continuous use of images is the choice in most cases. The robotic system generally includes an imaging system to obtain information of the operating target, a surgical actuator for the surgical operation, and a master end for control by the physician. For thoracic and abdominal surgery, the imaging system can obtain a clearer field of view (FOV) through stereoscopic vision and higher magnification due to the large operation space; typical examples are the da Vinci Surgical System of Intuitive Surgical Inc. (California, USA) [130,168], Microhand-S Surgical Robot of WeiGao Surgical Robot Ltd. (Shandong, China) [169], Toumai Endoscopic Surgery Robot of MicroPort Medical (Group) Ltd. (Shanghai, China) [170], and SHURUI Single-Port Endoscopic Surgery Robot of ShuRui Technology Ltd. (Beijing, China) [171]. In addition, some surgical robotic systems also incorporate VR technology to achieve visual guidance through 3D glasses or head-mounted display (HMD), reducing device size and improving user experience, such as Senhance Surgical System of Asensus Surgical Inc. (North Carolina, USA) [172] and Versius Surgical Robotic System of CMR Surgical Ltd. (Cambridge, UK) [173,174].

However, laparoscopic surgical robotics also presents several shortcomings. For example, the operating rooms for robot-assisted thoracic and abdominal surgery require special configuration due to the large size and space occupied by equipment. The complex equipment is prone to failure and requires frequent maintenance. Preoperative preparation and intraoperative instrument replacement are time-consuming, which also increases the burden for surgeons. With the ever-increasing trend of minimally invasive laparoscopic surgery, the volume allowing for visual devices is becoming increasingly limited, and thus miniature high-definition image sensors are required [31]. Furthermore, due to the limited field of view (FOV) of endoscopes, image enhancement and multi-mode image fusion algorithms are effective methods to increase the perception range of operators [109,125], which can also improve the success rate and safety of surgery. Due to the complexity of soft tissue deformation, deformable registration and tracking technology has not yet reached the requirement of surgical accuracy [175,176]. Existing laparoscopic robotic systems primarily operate through a master-slave control mode. The improvement of autonomy and intelligence through image processing technology remains a great challenge in the clinical application of such types of robotic systems [177–179].

### Orthopedic surgical robotics

Most orthopedic surgical robotic systems use preoperative CT images to generate 3D virtual models, while only a few of them incorporate MRI images [180]. Direct acquisition and discontinuous use of images are common choices due to the radiographic image's visibility of the bone structures, while indirect acquisition and continuous image use are preferred for procedures requiring high accuracy and timeliness. This category encompasses two main types: robots for navigation and positioning, and robots for surgical operation. The mainstream way of robotics for navigation and positioning is to use preoperative CT or intraoperative CBCT for tissue reconstruction and surgical planning, and then use radiography images or optical tracking systems to detect instrument deviations during surgery, so as to achieve dynamic safety control and accurate positioning of the robotic arm. For instance, Renaissance Surgical Robot of Mazor Robotics Ltd. (Caesarea, Israel) [132,137], and Zoezen Orthopedic Surgery Robot of Zoezen Robot Ltd. (Suzhou, China) [181] have adopted radiography images for observation, characteristic to direct acquisition and discontinuous use of images, whereas Mazor X Stealth of Medtronic Inc. (Minnesota, USA) after acquiring Mazor Inc. [182], ROSA One (Spine) Surgical Robot of Zimmer Biomet Holdings Inc. (Indiana, USA) [183], Excelsius GPS of Globus Medical Inc. (Delaware, USA) [184], and TiRobot Orthopedic Robotic System of TINAVI Medical Technologies Ltd. (Beijing, China) [136] use optical navigators for instrument tracking, which is distinct to the indirect acquisition and continuous use of images. These robots primarily assist surgeons in accurately positioning surgical tools, with the actual surgical operation performed by surgeons. In contrast, robots for surgical procedures refer to a surgical robot system designed for a specific type of surgery. For example, for orthopedic surgery, systems such as Mako Smart Robotics of Stryker Corp. (Michigan, USA) for total hip arthroplasty (THA) [151] and RossumRobot Fracture Reduction Robotic System of RossumRobot Technology Ltd. (Beijing, China) for pelvic fracture reduction surgery have been developed [185].

Bone tissue is commonly treated as a rigid body in robotic systems exploitation due to its high density and small deformation. Hence, indirect tracking using optical markers is the mainstream approach in the existing robotic systems with regards to registration and navigation. However, the implantation of markers causes additional damage and extends the operation time [37]. Therefore, the marker-free registration and tracking method has attracted significant research interest for implementation in these types of robots [36]. Moreover, the existing orthopedic robotic systems focus only on simple orthopedic surgeries with limited participation. Broadening the application scenarios of robots and assisting clinicians to complete more complex surgical operations are also important development directions in this category.

### Vascular interventional surgical robotics

Vascular interventional robotic systems used in the treatment of heart, brain, or cardiovascular system related diseases, usually adopt the master-slave control mode. The main console is located in an isolated control room, while the drive unit is located near the operating table, shielding surgeons from radiation. Guided by DSA images, the end of the catheter advances through the blood vessels by stepper motor, magnetic field mechanical control, or other approaches, belonging to the direct acquisition and continuous use of images. This category includes the Sensei X Robotic Catheter System of Hansen Medical Inc. (Delaware, USA), CorPath GRX System of Corindus Inc. (Massachusetts, USA) [133], ALLVAS Intravascular Interventional Surgery Robot of OperateRobot Technology Ltd. (Shanghai, China), Genesis RMN System of Stereotaxis Inc. (Missouri, USA) [186], and R-One Robotic Assistance Platform of Robocath Inc. (Rouen, France) [187,188].

Intraoperative vascular images are usually obtained by DSA. However, 2D images often fail to accurately depict the vascular system due to vascular overlap and fluoroscopic effects. Surgeons can rely only on subjective experience based on anatomy for 3D vascular reconstruction, resulting in prolonged operation time and lack of safety [189]. Therefore, the urgent implementation of 3D vascular reconstruction and intraoperative visualization technology with the assistance of magnetic resonance angiography (MRA) and computer tomography angiography (CTA), is necessary in the clinical application of such robotic systems [156,190,191].

### Puncture surgical robotics

Puncture surgical robots perform minimally invasive high-accuracy and high-efficiency puncture procedures for biopsy sampling or targeted therapy, including punctures of soft tissues, such as the lungs, liver, kidneys, and prostate, as well as of hard tissues, for example in neurosurgery. To perform punctures with minimal deformation and physiological movement, markers are usually attached to patients, robots, and intraoperative imaging equipment, and synchronous tracking through infrared, electromagnetic, visible light, and other trackers is performed for closed-loop control of robot action and achieve immediate error correction. Examples of such robots include the Epione Robotic System of Quantum Surgical (Montpellier, France), ROSA One (Brain) Surgical Robot of Zimmer Biomet Holdings Inc. (Indiana, USA) [135], and Remebot Neurosurgery Robotic System of Remebot Technology Ltd. (Beijing, China) [192], belonging to the indirect acquisition and continuous use of image category. For puncture surgery with large tissue deformation or with significant influence by physiological motion, the method of radiography image guidance is usually used in robot systems, such as XACT ACE Robotic System of XACT Robotics Inc. (Massachusetts, USA) [193], Soteria RCM Remote Controlled Manipulator of Soteria Medical (Arnhem, The Netherlands), Maxio Robotic System of Perfint Healthcare Pvt. Ltd. (Tamilnadu, India) [194], and Neuromate Stereotactic Robot of Renishaw plc (Gloucestershire, UK) [195,196], belonging to the direct acquisition and discontinuous use of images category.

Similar to orthopedic positioning and navigation robots, the robotic system using attached markers has the problems of additional patient tissue damage and prolonged operation time. Novel tracking techniques that provide intraoperative guidance and accurately fuse multimodality intraoperative information on tissue deformation and physiological motion tracking can effectively improve the operation accuracy of these types of robots [112–114]. On the other hand, radiography image-guided methods are limited by the imaging rate and have radiation problems. Clinical research on the application of non-radiation imaging techniques such as ultrasound image-guided puncture surgery have been conducted [197–199]. The integration of real-time tracking and navigation methods of non-radiation imaging into robotic systems is one of the future directions for development in this category [154,200].

### Natural orifice transluminal surgical robotics

Natural orifice transluminal surgical robotics refers to a robot that enters the human body through natural cavities, such as the throat, bronchi, urethra, stomach, and straight colon for diagnosis and surgery. These types of robots, which belong to the direct acquisition and continuous use of images category, usually integrate micro-vision sensors for image acquisition. Compared with the traditional endoscopic surgical system, the surgical instrument has better flexibility, can expand the surgeon's surgical ability, and play a role in reducing hand trembling. Their main application is for respiratory tract surgeries, such as Monarch Platform of Auris Health Inc. (California, USA) [164] and Ion Endoluminal System of Intuitive Surgical Inc. (California, USA) [201], and robots for alimentary canal surgeries like Flex Robotic System of MedRobotics Corp. (Massachusetts, USA) [202] and EndoLuminal Surgical System of ColubrisMX Inc. (Texas, USA) [203].

The operating space of this category of robots is narrower than that of laparoscopic surgical robots, especially during the process of going deep into human body tissues, which further reduces the operating space. Presently, 2D vision is the primary choice; however, these types of robots face problems such as high requirements for miniature high-definition sensors and effective stereoscopic visualization systems [204,159,205]. In addition, the complex network structure of human body cavities poses challenges for precise positioning as the robot advances through them in natural orifice transluminal surgical robotics [206–208].

### Other types of surgical robotics

Besides the main categories mentioned above, there are several other types with specific applications. For example, magnetically controlled capsule gastroscopy controls the movement of the robot through a magnetic field and integrates an image sensor for observation [209–213]; it belongs to the category of direct acquisition and continuous use. Implantable robotics stimulate tissue growth by applying traction force to dysplastic tissue and monitors the tissue growth process through radiography images [214]; it belongs to the direct acquisition and discontinuous use of images category. Otologic surgical robots for cochlear implantation use an endoscope for image guidance [215]; they belong to the direct acquisition and continuous use of images category. Ophthalmic surgical robotics is employed for treatment of fundus diseases, such as macular degeneration, glaucoma, and cataract; it injects drugs into the vitreous body of the eye under the observation of a microscope [216] and belongs to the direct acquisition and continuous use of images category. Tooth planting robotics performs photoelectric navigation for real-time positioning of the robot arm [134]; it belongs to the indirect acquisition and continuous use of images category.

Typical surgical robotics in current research fields and clinical practice are listed in Table 2, categorized based on the proposed classification method for image application. Laparoscopic surgical robotics and natural orifice transluminal surgical robotics belong to the direct acquisition and continuous/intermittent continuous use of images category, since the application environment of these kinds of robots is a transparent cavity; these robots can have integrated image sensors for real-time image acquisition. Vascular interventional surgical robotics also belongs to the direct acquisition and continuous use of images category, since the surgeon needs DSA to observe the posture of the instrument during the whole operation. Orthopedic surgical robotics and soft tissue percutaneous puncture surgical robotics belong to the direct acquisition and discontinuous use of images category, since the surgical procedures applied by these robots require high accuracy, and the target tissues are visible only through fluoroscopy. Robots that adopt markers for navigation belong to the indirect acquisition and continuous use of images category, as their target tissues are significantly affected by physiological movement, which puts forward higher requirements for the accuracy and real-time tracking of the target tissue and surgical instruments.

**Table 2.** Typical surgical robotics and classification.

			Classification				
Type	Name	Characteristics in Image Application	Direct	Indirect	Continuous	Intermittent Continuous	Dis-continuous
Laparoscopic surgical robotics	da Vinci Surgical Robot	Laparoscope & Fluorescence camera	●		●	●	
	Senhance Surgical System	Laparoscope	●		●		
	Versius Surgical Robotic System	Laparoscope	●		●		
	SHURUI Single-Port Endoscopic Surgery Robot	Laparoscope	●		●		
Orthopedic surgical robotics	TiRobot Orthopedic Robotic System	Preoperative CT & Intraoperative optical navigation		●	●		
	Renaissance Surgical Robot	Preoperative CT & Intraoperative registration with fluoroscopy by C-arm	●				●
	Zoezen Orthopedic Surgery Robot	Preoperative CT & Intraoperative 2D axial view after registration	●				●
	ROSA One (Spine) Surgical Robot	Preoperative CT & Intraoperative optical navigation		●	●		
	Mako Smart Robotics	Preoperative CT & Intraoperative optical navigation		●	●		
	RossumRobot Fracture Reduction Robotic System	Preoperative CT & Intraoperative optical navigation		●	●		
Vascular interven-tional surgical robotics	Sensei X Robotic Catheter System	DSA & 3D visualization module	●		●		
	Genesis RMN System	DSA	●		●		
	R-One Robotic Assistance Platform	DSA	●		●		
Puncture surgical robotics	ROSA One (Brain) Surgical Robot	Laser scanning registration & Optical navigation		●	●		
	Remebot Neurosurgery Robotic System	Visual marker navigation		●	●		
	XACT ACE Robotic System	Fluoroscopy image guidance	●				●
	Soteria RCM Remote Controlled Manipulator	MRI image guidance	●				●
	Neuromate Stereotactic Robot	Fluoroscopy image guidance	●				●
Natural orifice trans-luminal surgical robotics	Monarch Platform	Endoscopic image guidance	●		●		
	Ion Endoluminal System	Endoscopic image guidance	●		●		
	Flex Robotic System	Endoscopic image guidance	●		●		
Other surgical robotics	Capsule gastroscopy robotics	Camera & Wireless image transmission	●		●		
	Implantable robotics that stimulates tissue growth	Fluoroscopy image monitoring	●				●
	Otologic surgical robotics	Endoscopic image guidance	●		●		
	Ophthalmic surgical robotics	Image guidance under microscope	●		●		
	Tooth planting robotics	Preoperative CT & Intraoperative optical navigation		●	●		

## DISCUSSION AND CONCLUSION

Surgical robots possess technical capabilities surpassing those of humans, enabling precise operations with significant research and clinical application values. However, the completion of these operations and the progress of intelligent surgery largely depend on the fine perception of the surgical environment, especially the acquisition of images and the target-tracking method, making perception a quite appropriate classification criterion. This paper summarizes the application of images in surgical robotics and proposes a new category system dividing image application into two dimensions: direct/indirect and continuous/intermittent continuous/discontinuous. Here, we analyze the relationships and differences between the application mode and scenario for each category, aiming to provide a reference, guidance, and inspiration for future research on surgical robotics.

Regarding the types of imaging methods, the existing technologies include CT, MRI, ultrasound, laparoscopy, etc., which can present images of human tissues in a non-invasive way and provide guidance for surgical robotic systems. However, there is currently no balance between imaging resolution and time, necessitating the development of novel imaging technologies. In recent years, medical image processing technology based on deep learning has developed rapidly [217–219], and the reconstruction and visualization of human tissues has been widely used in clinical practice. Nevertheless, the reconstruction algorithm and accuracy of tiny tissues still need to be further explored [84,85,220]. Moreover, the emergence of 4D imaging systems (such as 4D CT and 4D MRI) enables the tracking of the spatiotemporal motion of organs during free breathing [221–224], which has a good application prospect. VR/AR technology development provides interactive surgical simulation possibilities, allowing clinicians to simulate surgical processes and obtain real-time feedback [225–228], which can effectively improve the efficiency of surgical planning and the success rate of surgery. Meanwhile, it can also reduce the clinician training time and training costs. Most surgical robotic systems rely on markers for real-time tracking, and the objects to be tracked are assumed to be rigid bodies. However, the process of implanting markers may cause additional damage to tissues, and the rigid body assumption is not valid in many cases either. Markerless tracking and deformation tracking will remain to attract research and clinical interest both in the present and future [175,176,229].

The acquisition and processing of images is an important part of the robot's perception of the environment, playing an irreplaceable role in the precise guidance of the robotic system. Surgical robots are different in design, function, target object, and procedures environment; furthermore, the image application mode is different. For operations that require better accuracy and visualization effects, direct imaging is needed, such as in laparoscopic operations and precise puncture treatment, whereas for procedures that pursue minimally invasive or inconvenience with direct image acquisition and processing, indirect image is better, such as positioning needle implantation and respiratory motion curve estimation. For procedures that need to reduce the dose of ionizing radiation, imaging is to be used discontinuously, as is the case in fracture reduction and drilling osteotomy, while for procedures with high dynamic tracking requirements, continuous use of image data is necessary, such as soft tissue punctures and neurosurgery. As one of the core algorithms for surgical robotic systems, researchers have put a lot of effort to solve the problem of medical image registration, but it is still difficult to achieve a fast and accurate registration scheme that meets clinical demands. The complexity and high accuracy requirements of human tissue deformation models underscore the urgent need for high-precision elastic registration solutions in the near future [109].

Direct imaging and/or marker-tracking methods are usually introduced to reduce spatial and/or temporal errors [147,230]. Indirect acquisition and continuous use of images usually adopts optical, electromagnetic, and other real-time navigators to track the location and position of markers, so as to indirectly realize real-time tracking in the surgical area or target tissue [114,117]. However, the process of marker implantation causes additional damage and prolongs the operation time, making the registration and tracking without markers a research hotspot in these types of robotic systems [36]. Direct acquisition and discontinuous use of images usually adopts radiography images, but real-time tracking fused with non-radiation imaging presents a difficult problem to solve in this category [154,200,231]. Direct acquisition and continuous use usually refer to laparoscopic or ultrasound image guidance, which has the problem of limited FOV. Direct acquisition and intermittent continuous use usually refer to laparoscopic ultrasound and fluorescence imaging, which can provide complementary information for the laparoscope. Therefore, image enhancement and multi-mode image fusion to increase the perception range is one of the development directions [109,125,232]. Certainly, the application mode mentioned above is not absolute, and guidance for surgical robotics can also be provided through a combination of various methods. In future research, we think, it's a good way to perform a multi-aspect analysis of the surgical demands, including important points, such as target tissues, application environment, operation characteristics, and functional requirements, putting it in a corresponding category as proposed in this paper. Then, taking the attributes of different image types (such as 2D/3D, shallow/deep information, with/without occlusion problem, etc.) into consideration, selecting appropriate positioning, orientation or guidance methods based on imaging, so as to promote the further development of medical, especially surgical, robotic systems.

**Figure 4.**
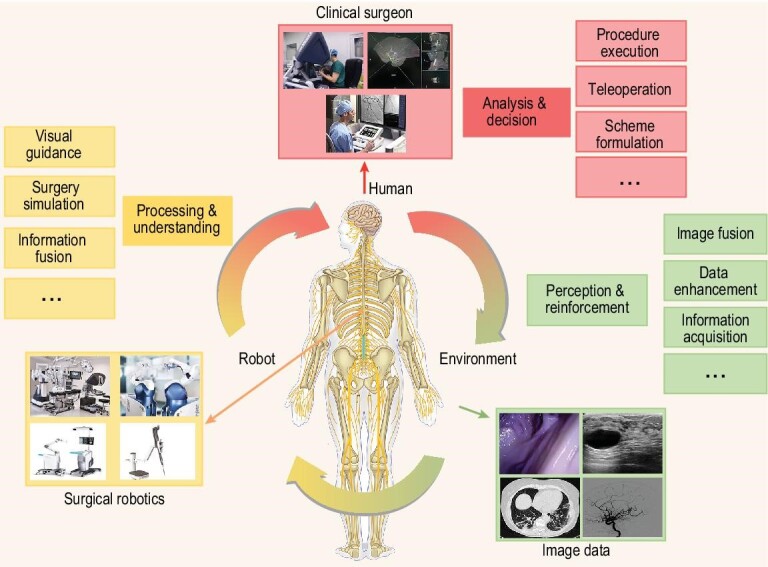
Surgical robot operation logic based on ‘Human-Robot-Environment’ integration.

In addition, establishing correlations and mapping between different spaces, including virtual image space, patient physical space, and robot motion space based on images, and the subsequent formulation of the execution logic is the basis for the realization of the function of surgical robotic systems. The central logic of the existing surgical robotic systems can be divided mainly into two categories: ‘human logic’ and ‘robot logic.’ For example, a robot with a master-slave control mode is human-centered, whereas the positioning robot is dominated by a computer algorithm. These two types of logic are in separate relationships in existing surgical robotic systems. The core of robots with ‘human logic’ operates in a logic close to that of traditional surgical methods, and can fuse clinicians’ experience and decision making directly and continuously. For example, in the da Vinci Surgical Robot, stereo-vision and master-slave operation and other methods that imitate people's logic have been adopted, which allows for conformable adjustments to the operation habits of surgeons, and thus it is easier to be accepted by them, but is quite deficient from an automation point of view. On the contrary, the advantages of robots with ‘robot logic’ as the core are mainly reflected in the automation, but the clinical experience and response decision of the surgeons can only be integrated into the robot system in an intermittent way. For example, the positioning process of an orthopedic robot is realized through clinicians’ planning and the intraoperative autonomous movement of the robot. ‘Human logic’ is excluded from the movement system in the process of continuous movement of the robot, making human participation insufficient. In the future, confronted with complex surgical environments, the integration of human and robot operation logic is expected to become an important development direction. We could draw an analogy with the human nervous system: the nerve terminal acquires information, the spinal cord processes basic logic such as stress reflex and ethical judgment, and the brain processes high-level logic such as emotional logic and multi-factor analysis decision making. In a surgical robotic system, images act as information perception; the robot processes and understands the information, and the human performs control at the highest level for analysis and decision making, as depicted in Fig. 4. Furthermore, with the continuous development of intelligence levels of robotic systems, their capabilities will progress from low- to high-level tasks, and human authority can also evolve to higher dimensions. Therefore, in the future development of surgical robotics, they can gradually advance further to perform more complex tasks from the perspective of functionality, significantly enhancing the intelligence and automation of surgical robotics [233].
